# Hints on the Management of Infants

**Published:** 1842-11-26

**Authors:** 


*Hints on the Management of Infants.*—There is so much good sense in a great part of M. Donné's work, that we have been tempted to make several extracts from it, or rather to put upon paper our own ideas on the same subjects, and blend them with those of our author. There may be no novelty in them; but they are not for this reason the less useful.

Let us, for the present, take one point connected with the *feeding of infants.*

Whatever be the age of the infant, it is always of great utility that the food be given at regular periods, as nearly as possible equi-distant from each other, so that the stomach of the young creature is neither left too long empty, nor oppressed and unnecessarily loaded. A sufficient interval of time must therefore be allowed to elapse after each repast, to enable it to be digested and then discharged from the stomach, before another be given. To put fresh food into the stomach, while it is still more or less filled with what had been taken before, is inevitably to disturb the process of assimilation, by which the food is converted into chyle, for the nourishment and growth of the body.

Now this very simple circumstance—so simple that the experience of daily life dictates it, and withal so important that the health cannot be maintained without due attention to it—is, strange to say, very seldom attended to in the rearing of infants. How generally do we observe, that instead of giving food to them only at regular and stated periods, no sooner do they begin to cry, than they are immediately applied to the breast, although, perhaps, not half an hour had elapsed since they had had it to their little heart's content. It surely needs not the authority of a medical man to assure any one that such a practice cannot be wholesome or right.

We might derive a useful lesson on this point by watching the conduct of the lower animals towards their young. We do not, for example, observe that the cow will allow the calf, or the sheep the lamb, to be tugging at the teat, whenever the young creature seems to wish it. It is only now and then, and at certain intervals, that they are allowed to satisfy themselves with the "milky food." We may be assured that nearly the same law applies to all animals, and that what holds good in the case of the calf and of the lamb, holds good of the human infant also.

In one respect only have the dumb creatures in our fields the advantage over us, and it is this: with them, *instinct*—that truly marvellous gift of an all-wise and beneficent Creator—is an unerring guide, while man's boasted *reason* is, alas! often either asleep, or is leading him astray.

But it may be asked, how often then should the infant have the breast? The following few directions will, we think, suffice to direct our readers on this very important question. The younger that the infant is, the more frequent is the desire, and necessity for food. During the first weeks of life, the breast may be given every two hours or so in the day—less frequently at night. As to the quantity which the child should have at each time, the nurse will, as a general remark, find that it may be allowed to continue sucking until it is satisfied. When once this is the case, all that is given afterwards is only oppressive to the stomach; and it is well known that the little creature usually gets rid of the superfluity by bringing it up. When the child is very weak, it may be necessary to give the milk more frequently, and in smaller quantities at a time; but in ordinary cases an interval of about two or three hours should be allowed to elapse between each meal.

The child should, however, seldom be awaked for the purpose of giving food. It is an old saying, that "a sound sleep is almost as nourishing as a full feast;" and the remark is especially true of infants. They require a great deal of sleep, and they are always refreshed and strengthened by it. As long as a child sleeps, we may rest pretty well assured that nature does not need any fresh supply of food; and therefore to disturb it merely because the regular period, at which otherwise it would have been applied to the breast, has arrived, is not only unnecessary, but hurtful. The exceptions to this remark occur very rarely indeed, and then only in the case of infants who are exceedingly weakly, and who should therefore be regularly seen by a medical man, as no general rule can be laid down that will be applicable to all.

It may be worth mentioning that, in not a few instances, the power of the nurse's milk seems to have something to do with excessive drowsiness of the infant. Nature appears to endeavour to compensate for the deficiency of nourishment by an extra allowance of repose. In all such cases, therefore, the medical man should satisfy himself most accurately on this point, more especially when the child is suckled by a hired nurse—who, it may be supposed, always tries to make us believe that her milk is both abundant and good. There is a simple rule which will very seldom misguide us, it is this: if the child does not thrive properly, and yet sleeps a great deal, we may feel pretty confident that the food administered is not sufficiently nutritious, and therefore that some change in the diet is necessary.

The frequency of suckling should be diminished after the sixth or seventh week, or even earlier, in the case of robust, hearty children. Once every three hours is then quite sufficient, provided the milk is duly nourishing, and the infant be allowed to satisfy its craving at each time. At the end of the third month, the breast need not be given oftener than every four hours; and so on successively, an extra hour being added to each interval after every additional month or six weeks of life. By following this system, not only is the health of the infant best consulted, but the convenience and comfort of the nurse will be greatly promoted.

How many a mother, from an overflowing affection, devotes almost every hour of every day to watching over her beloved child! She is unwilling to leave her home for even a few hours, from fear of it suffering for want of the breast while she is away. Now this is just one of those errors, which it is the leading object of M. Donné's work to endeavour to get rid of, both for the mother's and the child's sake. If the latter be accustomed from its birth to have food only at stated regular times, it is altogether better, in every respect, that in the intervals the nurse be away, and the child be left in the care of another person. We have already explained how much the condition of the milk is affected by the state of the general health, and how necessary it is for the maintenance of this, that the nurse should take regular out-door exercise, and that her mind be kept in an equably cheerful state. Now, how can these advantages be obtained, if she be, continually, from morning to night, with her child? Another great evil of the nurse being too much with the infant, is, that the latter, by the mere smell of the milk being constantly presented to it, is often induced to desire food when it certainly does not require it, and when, therefore, it would be much better without it.

This, it has always appeared to us, is one of the greatest disadvantages attending the nurse being too much with the infant—at least until it has been accustomed not to seek for the breast, except at stated intervals. And nature is so flexible, at this early period of life, that this most desirable end may easily be gained by adopting a judicious plan from the commencement.—*Med. Chir. Rev.* October, 1842.

